# Plastic impurities in biowaste treatment: environmental and economic life cycle assessment of a composting plant

**DOI:** 10.1007/s11356-023-28353-8

**Published:** 2023-07-05

**Authors:** Sara Bottausci, Chiara Magrini, Giulia Adele Tuci, Alessandra Bonoli

**Affiliations:** 1 Department of Civil, Chemical, Environmental and Materials Engineering, University of Bologna, 40131 Bologna, Italy; 2 Department of Environmental Science, Informatics and Statistics, University of Venice Ca’ Foscari, 30172 Venice Mestre, Italy

**Keywords:** Compostable plastic, Waste management, Life cycle costing, Material flow analysis, Food waste composting plant

## Abstract

The study focuses on an Italian composting plant and aims to investigate the impact of the presence of plastic impurities in the collected biowaste on the environmental and economic performance of the plant. The study is divided into two main steps: firstly, a material flow analysis was conducted to quantify the number of impurities (e.g., conventional plastics and compostable plastics) before and after the composting process. Secondly, a life cycle assessment (LCA) and a complementary life cycle costing (LCC) of the composting process were conducted. The results of the material flow analysis confirmed the initial assumption that conventional plastic remains almost constant before and after the composting treatment, while compostable plastic almost disappears. As far as the life cycle analyses are concerned, the most environmentally damaging phases of the process were the shredding and mixing phases, while the operating costs (OPEX) contributed the most to the total annual costs of the company. Finally, a further scenario analysis was performed, assuming that the plastic contaminants in the treated biowaste consisted exclusively of compostable plastics. The comparison with this ideal scenario can support decision-makers to understand the potential improvements achievable by addressing the presence of plastic impurities in the biowaste. The results show that the treatment of plastic impurities causes relevant environmental and economic impacts, being responsible for 46% of the total waste to treat at the end of the process, almost 7% of the total annual costs covered by the plant owners, and about 30% of all negative externalities.

**Supplementary Information:**

The online version contains supplementary material available at 10.1007/s11356-023-28353-8.

## Introduction

Circular economy practices are of utmost importance to strive for a more sustainable and resource-efficient world (Brandao et al. 2021). To close biological material cycles and reduce the linear economy of landfilling and incineration, it is crucial to properly treat organic waste. Indeed, it represents a considerable amount of municipal solid waste, i.e., 39.5% in Italy (ISPRA 2020) and an average of 34% in Europe (EEA 2020).

Composting is considered a fairly sustainable solution, as it significantly reduces the amount of waste that would otherwise go untreated (Pergola et al. 2018). Several studies have proven that composting facilities are extremely beneficial if the composting process is carried out correctly and the leachate is properly treated (Bernstad and la Cour Jansen 2011, Amlinger et al. 2008). Moreover, the compost produced in a composting plant is a high-quality, marketable product, suitable for small and large applications (Amlinger et al. 2008). As a consequence, composting is considered preferable compared to other waste management alternatives (Oliveira et al. 2017; Shen et al. 2021; Scoton et al. 2016).

Nevertheless, industrial compost production is still an environmentally damaging process due to the transport and several energy-intensive operations involved. In this context, previous studies (Amlinger et al. 2008; Boldrin et al. 2009) have addressed the problem of greenhouse gasses produced by fossil fuel-based transport and aerobic treatment. Moreover, when incorrect waste separation occurs, another problematic aspect is the presence of plastic contaminants that get into the organic fraction. In this case, the compost may be compromised, and the quality affected.

More specifically, conventional plastics do not degrade in the composting process and therefore, need to be separated, when possible, from the mature mixture (Gómez and Frederick 2013, Vaverková et al. 2014; Adamcová et al. 2013, 2017; Bandini et al. 2020; Alassalia et al. 2018). Additionally, composting processes cause conventional plastic products to fragment and become part of the compost that is eventually used as organic fertilizer in agriculture. This leads to the unintentional release of microplastics into the environment (Millican and Agarwal 2021, Braun et al. 2021). Research by (Braun et al. 2021) also found that the presence in composting facilities of microplastics from conventional plastics, e.g., PE and PVC, contributes significantly to greenhouse gas and ammonia emissions. Larger plastic products that can be separated from compost still pose a problem, as they cannot be recycled anymore but have to be sent to incinerators.

On the other hand, a positive trend has been observed in Italy for—compostable plastics (certified according to the national standard UNI 13432), whose presence in organic waste is increasing in 2019 compared to 2016–2017, resulting in an increment of 2.2% (COREPLA 2020).

The results of the surveys conducted between 2016 and 2019 by the Italian consortium COREPLA showed that the average product purity of the Organic Fraction of Municipal Solid Waste exceeds 95%. This means that less than 5% of the material delivered to biological treatment plants is non-compostable. Focusing on plastics, it has been observed that compostable plastics represent 1.4% of the material delivered, while conventional plastics represent 3.1%. In Italy, most of the bioplastic bags currently employed for food waste collection is made of the starch-based Mater-Bi polymer whose composition is 70% polybutylene adipate terephthalate, 20% starch, and 10% additives (Dolci et al. 2021).

Compostable bioplastics could provide a partial solution to this pressing problem by contributing to a real transition toward a circular bioeconomy (Beltran et al. 2021, Morone and Imbert 2020).

Studies have shown that compostable bioplastics decompose rapidly when treated appropriately (Gómez and Frederick 2013, Vaverková et al. 2014; Adamcová et al. 2017, 2013; Bandini et al. 2020; Alassalia et al. 2018). They can even increase the temperature and, thus, the decomposition of organic matter in compost (Sun et al. 2020).

Within this study, two follow-up analyses were developed to investigate the sustainability of a composting plant in the Emilia-Romagna region, Italy. First, a material flow analysis was conducted to quantify the number of impurities (e.g., conventional plastics and compostable plastics) before and after the composting process. Secondly, the main environmental and economic factors were identified through a life cycle assessment and life cycle cost analyses. Issues related to the presence of plastics in composting plants have been addressed by multiple researchers. However, none of the previous studies has attempted to combine a waste flow analysis with the application of an environmental and economic life cycle analysis. The novelty of this project is the enrichment of the research with two specific assessments that allow the comparison of two possible scenarios: the current condition, in which the waste scenario consists of the treatment of both conventional and compostable plastic, and an ideal scenario, in which the plastic waste fraction entering the process is ideally composed of only compostable plastic, which, thus, becomes part of the compost at the end of the treatment.

Life cycle analysis is indeed also widely applied in the context of organic waste management, and several studies developed comparison analysis based on life cycle analysis (LCA) to find the best alternative among different end-of-life solutions (Lu et al. 2020; Andersen et al. 2012; Saer et al. 2013). Furthermore, several other research combined LCA studies with complementary life cycle costing (LCC) to assess and compare different waste management system solutions (Lam et al. 2018). Some focused on municipal solid waste (Song et al. 2013) (Woon and Lo 2014), some on food waste (Gómez and Frederick 2013), and some on other kinds of waste (Rocchetti et al. 2013) (Simon et al. 2013).

The development of LCC analysis for waste management systems is not yet as widespread as LCA, but it has nevertheless shown its impressive potential in decision-making processes (Li et al. 2021, Lu et al. 2020, Woon and Lo 2014, Lee et al. 2020, Dong et al. 2014, Elagroudy et al. 2011, Reich 2005).

The paper is structured in four sections. In the “Materials and methods” section, authors describe the methodologies applied to analyze the composting plant and the scenario analysis is explained. The “Results and discussion” section discusses the results, while in the “Conclusion” section, authors draw some conclusions.

## Materials and methods

In Italy, the organic fraction is mostly collected separately from the rest of the municipal waste and sent for proper treatment. According to (ISPRA 2020), in 2019, 49.2% of the collected organic waste was disposed of in composting plants, 45.7% was treated by integrated anaerobic–aerobic methods, and the remaining 5.1% was sent to anaerobic digestion. At regional government level, the Emilia-Romagna Region set an ambitious target on recycling of organic waste already in 2015, putting circular economy policies at the core of regional waste management planning (Magrini et al. 2021).

This case study concerns a composting facility managed by the cooperative “La Città Verde”, which collects urban waste from different Italian municipalities, a vast majority within the Region (see SI for details). Approximately 85% of the total amount of waste collected by the cooperative is municipal waste, the remaining part is special waste from production activities such as markets and agricultural industries.

The composting plant of La Città Verde is located in Crevalcore (Emilia-Romagna Region, Italy) and treats around 15,000 tons of biowaste every year to produce and commercialize compost, according to the Italian law 75/2010 requirements (La Città Verde n.d, ANPA 2002). It also produces lignocellulosic biomass from green waste (wood chips) to be used as biofuel. More information on the plant and the cooperative is described in the supplementary information (SI). The organic waste collected at La Città Verde is initially taken into a shed where it is placed in a mixer using an automated mechanical arm and then mixed with the lignocellulosic material.

The ratio between the two materials depends mainly on seasonality and, generally, it is about 60% of organic waste and 40% of lignocellulosic material. Besides seasonality, the material entering the plant also depends on some other variables such as: presence of dust, moisture and the quality of the woody structuring material. In any case, the pile is then treated in two distinct phases, regulated with different temperatures. The first phase is the mesophilic phase, which works within a temperature range between 18 and 44 °C. Most of the simplest organic substances, e.g., carbohydrates, lipids, and proteins, are transformed into water, heat, and CO_2_. In particular, heat is an essential element because of its bactericidal action. The piles are insufflated from the floor with air, whose flow rate can be controlled to regulate the pile temperature.

Subsequently, in the second thermophile phase, the heap is moved into a specific container having a maximum capacity of 250 tons and subject to temperature regulation. During the second phase, microorganisms grow and degrade other organic fractions through hydrolysis. The process lasts around 30 days: it ends when the heap loses the correct amount of water as indicated by Italian law (Legislative degree 1998). A considerable amount of leachate is generated during the process and transported to two treatment facilities. For what concerns the gas produced, all air emissions from the plant are conveyed through a mechanical biofilter that consequently does not need energy supply.

Afterward, during the curing phase, which lasts for 20 days, the compost maturates inside a particular container reaching a maximum temperature of 55 °C for the amount of days required by Italian law (Legislative degree 1998) in order to favor mesophilic bacteria that “produce” fulvic and humid acids. Multiple software can regulate temperature changes and percolate growth from the control cabin. Lastly, the compost is brought outside, where it matures for a further 40-day period in open cells, for a total of 90 days. In this final stage, the compost is stable and does not emit any odors.

The resulting compost is then sifted and managed according to the size. Composting products having less than 10 mm diameter are the actual compost, ready to be sold. The quality of the final product is obviously related to the quality of the input materials with respect to their degree of putrescence, cellulose and water content.

Before being sold the very final composting product is analyzed in batches at least twice a year and must meet the parameters to be defined as mixed composted soil according to the Italian decree 75/2010 on fertilizers (Legislative degree 2010). The products ranging between 10 and 50 mm in diameter that mainly consists of ligneous materials are reused in a new composting process with a new fresh organic fraction, this fraction consequently contains some plastic impurities that derive from the organic waste; however, the amount of these impurities is negligible compared to those present in the organic waste and was therefore not considered in this study. The residual waste (e.g., plastics and other non-organic materials) is sent to proper treatment.

### Waste flow analysis

A waste flow analysis can be conducted by using different methods. Compost sampling is the first step to performing a mass characterization analysis, in which different types of organic materials are involved.

The quartering method, as described in the UNI EN ISO no. 5667–13 (2011), was selected for this analysis, as it is a commonly used and recommended method to analyze and characterize municipal solid waste (Torres-Pereda et al. 2020; Drudi et al. 2015; Sudhir Kumar et al. 2020).

Firstly, the sample is mixed on a surface to form a cone. During this phase, it is recommended to drop the material from the top of the cone to guarantee proper distribution. Afterward, the heap is homogeneously divided into quarters; then, two quarters are selected and combined. This procedure is repeated until the last two quarters form the mass sample required.

The quartering method was applied following general guidelines (ARPA Piemonte 1998) and adapted to our case study which consists of a total amount of 1600 kg initial fresh organic waste.

After the quartering process, a waste flow analysis was performed to identify the amount of plastic and compostable plastic entering the plant and the amount remaining after the composting process through a visual inspection.

To this end, the fresh organic waste was first analyzed before being mixed with the lignocellulosic component, and then the mature mixing was examined before being screened. Subsequently, through a visual inspection, the obtained 115 kg of waste was laid on a table and sieved into four categories: organic waste, conventional plastic, compostable plastic, and other kinds of wastes, e.g., aluminum textiles, polyamines, paper, and other non-organic materials. The different groups were then weighted. In this phase, it was possible to note that compostable plastic bags represented the largest share of the compostable plastics, in line with the literature (Sailer et al. 2021).

The second analysis was performed on the mature compost before it was sifted. Considering that this mixture was already mixed several times throughout the whole process, 100 kg of material from the pile was selected and analyzed. The resulting sample was significantly homogenous, and therefore, only part of the sample (40 kg) was investigated. The material under analysis was laid on a table, manually sorted, and divided into plastic, compostable plastic, compost, and other waste. Lastly, all the separate groups were weighted.

### Life cycle assessment

LCA is a standardized method known and used worldwide (ISO 14040 2006a, ISO 14044 2006b). Because of its holistic approach, it represents a great support tool for decision-making processes, given the significant potential of avoiding burdens shifting problems.

The methodology allows the users to analyze simultaneously several environmental impact categories related to a product, a service, or even a system throughout the entire life cycle (European Commission 2010a, European Commission 2010b).

According to the official standard of environmental management ISO 14040:2006 (ISO 14040 2006a), the LCA methodology consists of four iterative steps: goal and scope definition, life cycle inventory, life cycle impact assessment, and interpretation of the results.

The assessment was developed by applying an attributional LCA through a cut-off approach and by using the SimaPro 8.5 software. According to the study purposes and the geographical area considered, the modeling was conducted by applying the CML-IA baseline methodology (Guinée et al. 2002).

The CML-IA baseline method does not take into account biogenic CO_2_ emissions in the calculations: this fits the context of the research. Indeed, several authors in the literature suggested not considering biogenic CO_2_ in the impact assessment of the composting process (Pergola et al. 2018, Amlinger et al. 2008, Boldrin et al. 2009, Saer et al. 2013, Bjarnadóttir et al. 2002, Chen and Lin 2008, Quirós et al. 2014, Zhao et al. 2009).

The life cycle assessment analysis was performed considering the sole composting process and the waste management system as the main focus of the study. Consequently, potential environmental impacts imputable to the operational activities of the rest of the facility, e.g., heating structure system, water consumed as a service, and workers’ equipment, were not considered.

The functional unit (FU) used is the total amount of food and wood waste delivered to La Città Verde in 2020 (15,000 tons, see Table 1 for more details). Data gathered were mainly primary data provided by the Waste Treatment Manager and adapted to our scope, guaranteeing a high adherence to reality. When needed, secondary data was gathered from the Ecoinvent v.3 databases (Wernet et al. 2016).

**Table 1:** Life cycle inventory, referred to as the functional unit (FU)

Element	Amount	Unit of measure	Data source
Transportation			
Input			
Organic waste	13,500	ton	Primary data
Wood waste	1500	ton	Primary data
Lorry 16–32 t	95,923.2	tkm	Primary data for transportation distances Ecoinvent database for impacts of the transportation mean (more details in SI)
Shredding mixing and maturation			
Input			
Collected waste (organic and wood)	15,000	ton	Primary data
Reused wood waste	3,000	ton	Primary data
Mechanical shovels	61.46	ton	Primary data for the consumption of the machine Ecoinvent scenario for the machine related impacts (more details in SI)
Mixer	228,800	kWh	Primary data for the consumption of the machine Ecoinvent scenario for the machine related impacts (more details in SI)
Aspirator	385,440	kWh	Primary data for the consumption of the machine Ecoinvent scenario for the machine related impacts (more details in SI)
Output			
Ammonia	0.04	ton	Primary data
Water (evapotranspiration)	0.04	ton	Primary data
Evaporation	2,608	ton	Primary data
Leachate to wastewater treatment	1200	ton	Primary data for the volume of the leachate Ecoinvent process for the wastewater treatment scenario (more details in SI)
Screening and waste treatment			
Input			
Mature compost	14,191.2	ton	Primary data
Screener	114,400	kWh	Primary data
Output			
Wood chips waste to sell	4430.5	ton	Primary data
Wood to reuse	3,000	ton	Primary data
Metal waste treatment	715.5	ton	Primary data for the volume of the waste Ecoinvent process for the metal waste treatment scenario (more details in SI)
Glass waste treatment		ton	Primary data for the volume of the waste Ecoinvent process for the glass waste treatment scenario (more details in SI)
Textile waste treatment		ton	Primary data for the volume of the waste Ecoinvent process for the textile waste treatment scenario (more details in SI)
Plastic mixture waste treatment	600	ton	Primary data for the volume of the waste Ecoinvent process for the plastic waste treatment scenario (more details in SI)
Final compost	5,446	ton	Primary data

As this study was based on the results of a single compositional analysis, the uncertainty in the input values could lead to a large variability in the LCA and LCC results. Uncertainty was not evaluated in this study.

A cradle-to-gate analysis was performed, setting the boundaries from the waste collection to the production of the final compost. Following the “zero burden” assumption, waste has no burdens before it becomes waste (Ekvall et al. 2007). Wood and organic waste are collected from several collection sites and transported to the composting plant. Subsequently, they are mixed and shredded with the residual wood waste from former compost productions. At this point, the product undergoes two maturation stages (indoor and outdoor), and lastly, it is sent to the screening phase, whose output is marketable compost. The diagram represents the maturation steps as part of the shredding and the mixing phase. There is also a supplementary step through which the residual wood material is again screened, and additional wood chip products are gained to be used for a new process. Other kinds of waste are inevitably produced and sent to external treatment facilities. Notably, almost the entire fraction of residual solid waste at the end of the treatment is composed of plastic, while smaller contributions are given by metal, glass, and textile waste. The residual plastic represents 4% of the total waste entering the plant and being treated. Additionally, during the first stage of the process, 8% of leachate is generated and is currently sent to external treatment facilities. The distribution of the compost to external consumers and its use is not included in the boundaries. Lastly, a total annual amount of 370 OuE/m^3^ odor emissions, well below the authorization limits, were identified and included in the LCI as part of the evaporation emissions, among which, ammonia represents the major contributor.

The life cycle inventory (LCI) is reported in Table 1 (see SI for further information).

### Life cycle cost analysis

Life cycle costing is an economic analysis frequently used to complement LCA in decision-oriented processes (Lu et al. 2020; Elagroudy et al. 2011) and calculate the costs along the life cycle of a product or a system (Hunkeler et al. 2008).

Within this case study, a societal LCC was performed, referring to the year 2020. Societal LCC can be defined as the assessment of all costs associated with the life cycle of a product directly covered by one or more of the actors in the product life cycle (supplier, manufacturer, the user or consumer, and/or end-of-life actor), with the inclusion of externalities that are anticipated to be internalized in the decision-relevant future (Hunkeler et al. 2008). The primary objective is to focus specifically on a financial analysis of the waste management system using the system boundary set out by the associated LCA study. Indeed, following Edwards et al. (2018) the boundaries and the functional unit used for the LCA analysis were maintained, while specifying the different actors involved (i.e., waste collectors who committed the waste in input to the plant, plant owners, waste managers responsible for the treatment of residual waste).

As far as waste collectors are concerned, the cost for the transport of waste to the plant is the only one considered in the analysis: it includes the costs for fuel, motorway tolls, and personnel costs (more details are available in SI).

The costs covered by the plant owners include operating costs and capital costs, while their incomes come from the waste in input committed to the plant and the sale of compost and wood chips.

On the one hand, the costs are primary data, since they were provided by the plant manager; on the other hand, some estimations were necessary to calculate the incomes from the waste committed to the plant: to estimate this value, first, all the contracts which the plant had with customers in 2018 were analyzed, in terms of the maximum amount of waste which each of them could yearly commit to the plant and the price per ton. A weighted average of the price per ton was calculated, both for organic waste and wood waste: this value was used to calculate a proxy value of the incomes. As far as incomes from the selling of compost are concerned, an average price equal to 3 €/ton was considered (primary data), while the unit average price of wood chips is equal to 20 €/ton (moisture content of less than 40% and particle size of up to 40 mm).

Finally, the cost for the treatment of residual waste in output was also included, as well as the cost for the treatment of the leachate.

Moreover, following Magrini et al. 2022 who studied the waste management system of the Emilia-Romagna Region, the externalities from the transportation of waste, the consumption of electricity of the plant and the incineration of residual waste were also calculated. The externalities from the incineration include both the external costs from the emission of macropollutants and the external benefits from the avoided electricity production. The externalities from the emissions of the composting plant were not included, considering that the CO_2_ is biogenic, and the use of the biofilter allows the reduction of VOC to a negligible level.

Societal LCC was already applied to a composting plant within the broader study conducted by Martinez-Sanchez et al. (2017), who estimated the externality costs based on emissions of some pollutants to air (CO_2_, CH_4_, N_2_O, PM_2.5_, PM_10_, NO_x_, SO_2_, VOC, CO, NH_3_, Hg, Pb, Cd, Cr (VI), Ni, As, and dioxins). Moreover, Edwards et al. (2018), who analyzed seven unique food waste management systems, incorporated both an Environmental LCC and Societal LCC, considering the externalities related to the emissions into the air and water. The approach followed in this paper differs from the two above mentioned, because only three specific aspects (i.e., transportation of waste, electricity consumption, and incineration of residual waste) were selected as interesting and therefore analyzed in depth.

Further details on the methodology followed for the assessment of externalities are described in the SI.

### Scenario analysis

To frame a more complete evaluation and to better tackle plastic-related issues concerning composting activities, the life cycle methodologies were applied to assess an ideal scenario. In this scenario, it was assumed that all the plastic in the input consisted of compostable plastic, which, therefore, became part of the compost at the end of the treatment without generating additional plastic waste residuals. This assumption is also supported by literature studies that developed specific works on the degradability of different kinds of bioplastic during the composting process (Ohtaki and Nakasaki 2000). In particular, for this case study, compostable plastic food packaging and compostable plastic bags, whose degradability conditions are suitable for composting, are of interest (Ohtaki and Nakasaki 2000). This scenario represents a condition in which the domestic waste is carefully sorted into the correct bins at home, resulting in high-quality organic waste, free from all the non-biodegradable residual waste.

Table 2 shows the revised inventory data with the assumption of considering only compostable plastic in the treatment and assuming the same prior functional unit equal to the total amount of food and wood waste delivered to La Città Verde in 2020.

**Table 2:** Life cycle inventory—scenario analysis (FU)

Element	Amount	Unit of measure	Data sources
Transportation			
Input			
Organic waste	13,500	ton	Primary data
Wood waste	1500	ton	Primary data
Lorry 16–32 t	95,923.2	tkm	Primary data for transportation distances Ecoinvent database for impacts of the transportation mean
Shredding mixing and maturation			
Input			
Collected waste (organic + wood)	15,000	ton	Primary data
Reused wood waste	3,000	ton	Primary data
Mechanical shovels	61.46	ton	Primary data for the consumption of the machine Ecoinvent scenario for the machine related impacts
Mixer	228,800	kWh	Primary data for the consumption of the machine Ecoinvent scenario for the machine related impacts
Aspirator	385,440	kWh	Primary data for the consumption of the machine Ecoinvent scenario for the machine related impacts
Output			
Ammonia	0.04	ton	Primary data
Water (evapotranspiration)	0.04	ton	Primary data
Evaporation	2,608	ton	Primary data
Leachate to wastewater treatment	1200	ton	Primary data for the volume of the leachate Ecoinvent process for the wastewater treatment scenario (more details in SI)
Screening and waste management			
Input			
Mature compost	14,191.2	ton	Primary data
Screener	114,400	kWh	Primary data for the consumption of the machine Ecoinvent scenario for the machine related impacts
Output			
Wood chips waste to sell	4430.5	ton	Primary data
Wood to reuse	3000	ton	Primary data
Metal waste treatment	715.5	ton	Primary data for the volume of the waste Ecoinvent process for the metal waste treatment scenario (more details in SI)
Glass waste treatment		ton	Primary data for the volume of the waste Ecoinvent process for the glass waste treatment scenario (more details in SI)
Textile waste treatment		ton	Primary data for the volume of the waste Ecoinvent process for the plastic waste treatment scenario (more details in SI)
Final compost	6,046	ton	Hypothetical scenario—assumption based on primary data

## Results and discussion

### Waste flow analysis results

The results of the waste flow analysis are shown in Table 3 and Table 4, for the fresh organic waste and the mature compost, respectively.

**Table 3:** Waste sample classification before the composting treatment

Type of waste	Mass (kg)	Percentage (%)
Organic biodegradable	101	87.8
Compostable plastic	5.5	4.78
Plastic	4	3.5
Other non-degradable waste	3	2.6
Total organic fresh waste	115	100
Loss	1.5	1.3

**Table 4:** Waste sample classification after the composting treatment

Type of waste	Mass (kg)	Percentage (%)
Organic biodegradable	36	90
Compostable plastic	0.250	0.63
Plastic	1.5	3.75
Other non-degradable waste	1.2	3
Total compost not sieved	40	100
Loss	1.05	2.63

As expected, the percentage of conventional plastics remains nearly constant before and after the treatment. On the other hand, compostable plastic almost disappears in the composted fraction of waste, consisting of only 0.63% of the total amount.

The organic waste analyzed resulted to be relatively homogenous: only a tiny fraction of the total fresh organic waste was extraneous material. This includes plastics and textiles, aluminum, and other materials in smaller shares. Compostable plastic products represent 4.8% of the total waste fraction.

It is worth noting that the compostable plastic found in the analyzed waste were only represented by compostable plastic bags, mainly made of Mater-bi, which were appropriately disposed of in the organic waste, whereas the other non-compostable materials, such as the conventional plastics, were wrongfully placed in the organic waste bin, either for lack of attention or lack of information on the correct disposal of the material.

From this analysis, it was possible to confirm that compostable bioplastic is indeed a good solution to plastic pollution issues if adequately managed. On the other hand, the analysis also confirmed that conventional plastic represents a severe problem in composting facilities due to its chemical characteristics. The results are consistent with other studies (Gómez and Frederick 2013, Vaverková et al. 2014; Adamcová et al. 2017, 2013; Bandini et al. 2020; Alassalia et al. 2018) and highlight the importance of a carefully sorted collection of biowaste to obtain high-quality compost through information campaign on the importance of proper domestic waste sorting. Cesaro et al. 2016, Hungría et al. 2017, and Li et al. 2016 also obtained similar results describing plastics as the major impurity fraction in the total mass entering the process ranging between 0.68 and 2.51% of fresh mass for urban areas and between 0.35% and 1.58% for rural areas.

**Fig. 1:**
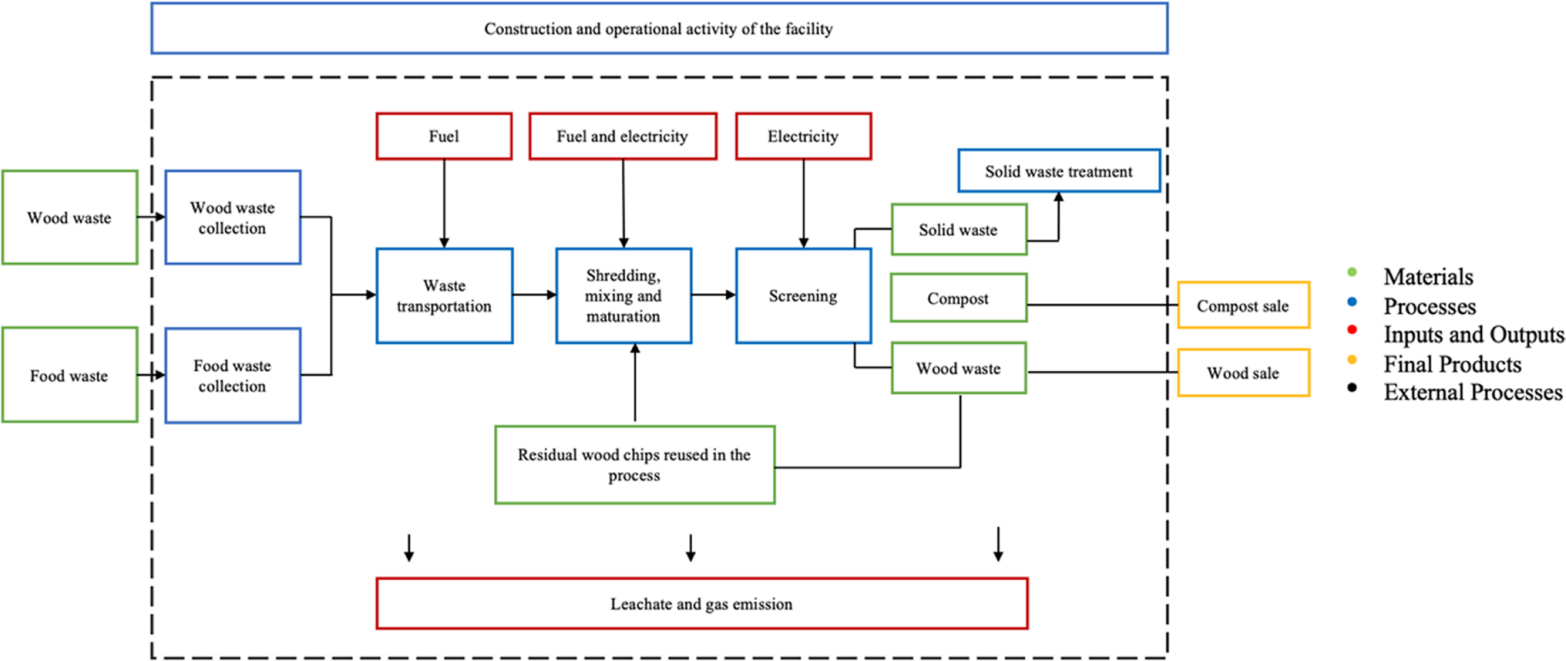
System boundaries (dashed line) of the LCA. Materials flows are represented in green squares, processes in blue ones (both inside and outside the system boundaries), and outputs and inputs in red color. The final products (compost and wood to sell) are shown in dark blue squares

On the other hand, Sailer et al. 2021 showed lower percentages at the end of the composting treatment because of a pre-treatment that can reduce impurities in outputs and deliver higher-quality compost (Fig. 1).

### LCA results and discussion

**Fig. 2:**
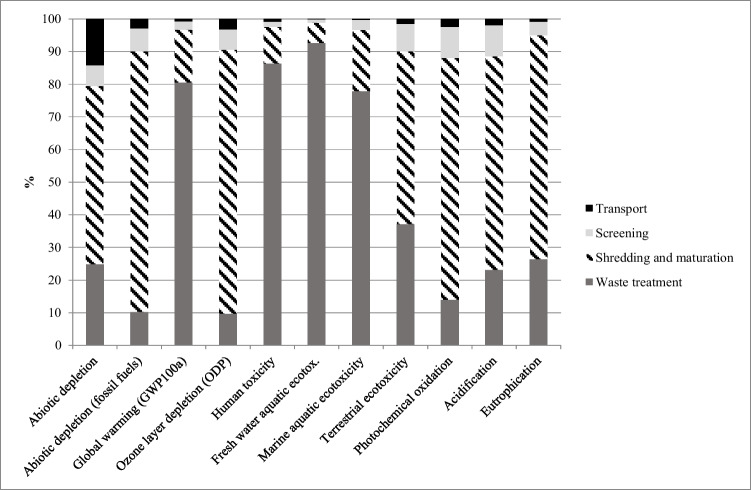
LCIA results per FU. A comparison representation of the impacts during the final waste treatment, the shredding and maturation step, the screening stage, and the initial transport of waste to the composting facility center

**Fig. 3:**
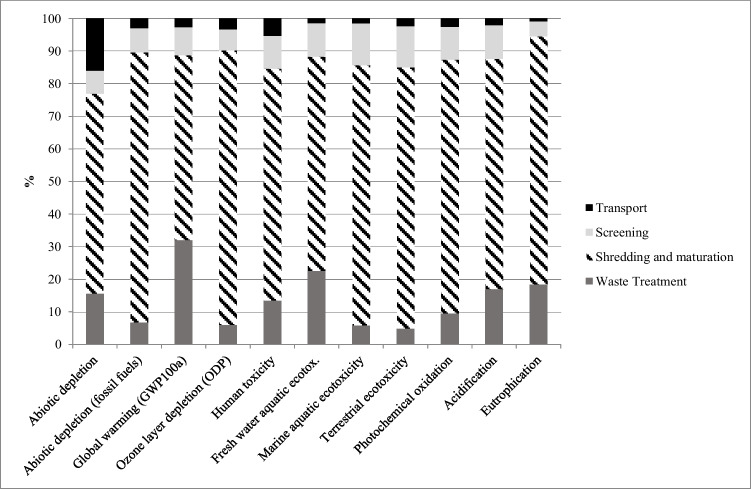
LCIA results per FU, scenario analysis. A comparison representation of the impacts during the final waste treatment, the shredding and maturation step, the screening stage, and the initial transport of waste to the composting facility

The LCIA results are reported in Fig. 2 as relative contribution grouped by three main phases of the process (i.e., screening, shredding and maturation, transport) and the waste treatment and in Table 5 and Fig. 3 as absolute values.

**Table 5:** Absolute results for FU and absolute results for 1 ton of compost obtained

		Functional unit results—our study	Results for 1 ton of compost—our study
Impact category	Unit	Total	Total
Abiotic depletion	kg Sb eq	9.24E − 01	1.70E − 04
Abiotic depletion (fossil fuels)	MJ	7.95E + 06	1.46E + 03
Global warming (GWP100a)	kg CO2 eq	1.98E + 06	3.63E + 02
Ozone layer depletion (ODP)	kg CFC-11 eq	8.71E − 02	1.60E − 05
Human toxicity	kg 1.4-DB eq	6.24E + 05	1.15E + 02
Fresh water aquatic ecotox	kg 1.4-DB eq	1.13E + 06	2.08E + 02
Marine aquatic ecotoxicity	kg 1.4-DB eq	1.28E + 09	2.35E + 05
Terrestrial ecotoxicity	kg 1.4-DB eq	1.27E + 03	2.34E − 01
Photochemical oxidation	kg C2H4 eq	8.85E + 01	1.62E − 02
Acidification	kg SO2 eq	2.54E + 03	4.67E − 01
Eutrophication	kg PO4–- eq	1.53E + 03	2.81E − 01

Analyzing, first, the relative contributions, it can be noted that the shredding and maturation phase is the main contributor in 7 out of the 11 impact categories analyzed. This is a reasonable result, considering the high energy consumption of these stages. This result is also consistent with the study developed by (Lu et al. 2020), in which energy consumption has the most significant impact. On the other hand, (Boldrin et al. 2009) and (Lundie and Peters 2005) stated that most environmental impacts were imputable to transportation and waste collection. This may be due to the type of transportation means used and the distance traveled. In our case, only local and regional collections were considered, and therefore relatively short distances were calculated.

Acidification and eutrophication potential indicators are significantly high in the shredding and mixing phase, and this is caused by the generation of leachate, and, to a lesser degree, also by some ammonia emissions, although the efficiency of the facility in treating and regulating ammonia emissions very high. Indeed, the emissions generated during the decomposition are beneath the legal threshold as a result of the use of a biofilter which constantly captures and biologically degrades pollutants. Similar results have also been demonstrated by (Saer et al. 2013), which developed an exhaustive attributional life cycle analysis of a food waste composting system with the novelty of considering the use of the compost as a soil conditioner and as an alternative to peat. (Saer et al. 2013) concluded that compost processing was the most environmentally demanding stage in the procedure due to decomposition emissions, which highly impact global warming, acidification, and eutrophication.

Marine aquatic, freshwater aquatic, and human ecotoxicity are primarily associated with the last stage of the process when the compost is finally ready, and the residual waste is separated and then incinerated in specific treatment facilities. The incineration of waste is, indeed, potentially harmful both for humans and for the environment.

Moving to the absolute results of the composting process studied, a comparison analysis with literature was developed and consistent outcomes were found.

The comparison was performed by normalizing the results to the FU used in literature (Table 5) which was 1 ton of compost obtained at the end of the process. It was found that Global Warming resulted in a total of 363 kg CO2 eq which is consistent with results obtained by Tonini et al. (Tonini et al. 2020), Mancini (Mancini et al. 2019) and Slorach (Slorach et al. 2019) (Table 6). Accordingly, ozone depletion outcomes (1.60E − 05 kg CFC-11 eq) are in accordance with di Maria (Di Maria et al. 2016) and with a more recent study by Slorach (Slorach et al. 2019). Freshwater ecotoxicity impacts result in a total of 208 kg 1.4-DB eq as seen in Saer (Saer et al 2013).

**Table 6:** Comparison analysis with literature studies, normalized to 1 ton of compost obtained at the end of the process. Adapted upon La Pera et al. (2022)

		FU results	Results for 1 ton of compost	Authors—results for 1 ton of compost									
		Our study		Sailer et. at	Tonini	Mancini	Slorach	Salemdeeb	Saer	Di Maria	Thyberg	Lombardi	Di Maria
Impact category	Unit	2021		2021	2020	2019	2019	2018	2013	2015	2017	2015	2016
Abiotic depletion	kg Sb eq	9.24E − 01	1.70E − 04										
Abiotic depletion (fossil fuels)	MJ	7.95E + 06	1.46E + 03										
Global warming (GWP100a)	kg CO2 eq	1.98E + 06	3.63E + 02	− 1.18E + 01	3. .50E + 02	4.35E + 02	2.58E + 02	1.52E + 01	2.21E + 05	1.37E + 03	+ 21; + 515	− 1.72E + 02	1.62E + 02
Ozone layer depletion (ODP)	kg CFC-11 eq	8.71E − 02	1.60E − 05	− 1.00E − 04	1.90E − 03	3.83E − 05	2.93E − 05	9.10E − 07	2.44E − 06	1.72E − 05	− 6.50E − 06	3.60E − 06	9.18E − 06
Human toxicity	kg 1.4-DB eq	6.24E + 05	1.15E + 02										
Fresh water aquatic ecotox	kg 1.4-DB eq	1.13E + 06	2.08E + 02	1.40E − 01	4.50E − 07	1.93E + 01	4.67E + 00	1.20E − 02	1.61E + 02	3.20E − 01			
Marine aquatic ecotoxicity	kg 1.4-DB eq	1.28E + 09	2.35E + 05	− 4.82E + 00	1.65E + 01	4.33E + 00							
Terrestrial ecotoxicity	kg 1.4-DB eq	1.27E + 03	2.34E − 01	2.71E + 01	7.00E − 02	1.30E − 02							
Photochemical oxidation	kg C2H4 eq	8.85E + 01	1.62E − 02										
Acidification	kg SO2 eq	2.54E + 03	4.67E − 01										
Eutrophication	kg PO4–- eq	1.53E + 03	2.81E − 01										

Considering the scenario analysis, as far as the waste treatment is concerned, the results obtained are significantly different. The presence of plastic in the residual solid waste at the end of the treatment has important implications in most of the impact categories analyzed as shown in Fig. 3.

More specifically, human, marine, and terrestrial ecotoxicity reduce, respectively, by 84.5%, 92.6%, and 87.3%. An impressive reduction rate also occurs for Global Warming whose impacts are inevitably related to incineration facilities for solid waste treatment. Further information about the reduction rates from the comparison of the two different scenarios is grouped and shown in SI.

**Fig. 4:**
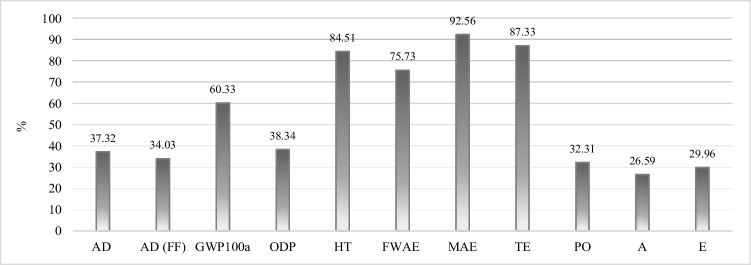
Percentage of reduction of the impacts of the waste treatment step between the baseline scenario relative contributions and the scenario analysis relative contributions (more details in SI). Results per FU. In particular, the acronyms stand for the following: AD (abiotic depletion), AD (FF) (abiotic depletion fossil fuel), GWP100a (global warming potential), human toxicity (HT), freshwater aquatic ecotoxicity (FWAE), marine aquatic ecotoxicity (MAE), terrestrial ecotoxicity (TE), photochemical oxidation (PO), acidification (A), eutrophication (E)

A more detailed difference regarding waste treatment (WT) is reported in Fig. 4, which compares the current scenario—WT *as-is*—in which plastic products are present in biowaste and consequently they undertake a treatment after the composting process, with an ideal scenario—WT *to-be*—in which only compostable plastic was assumed present in the collected biowaste and, therefore, able to degrade and to turn into compost at the end of the treatment process. Additional details on the percentage of reduction from the scenario *as is* to the scenario *to be* in SI.

### LCC results and discussion

**Fig. 5:**
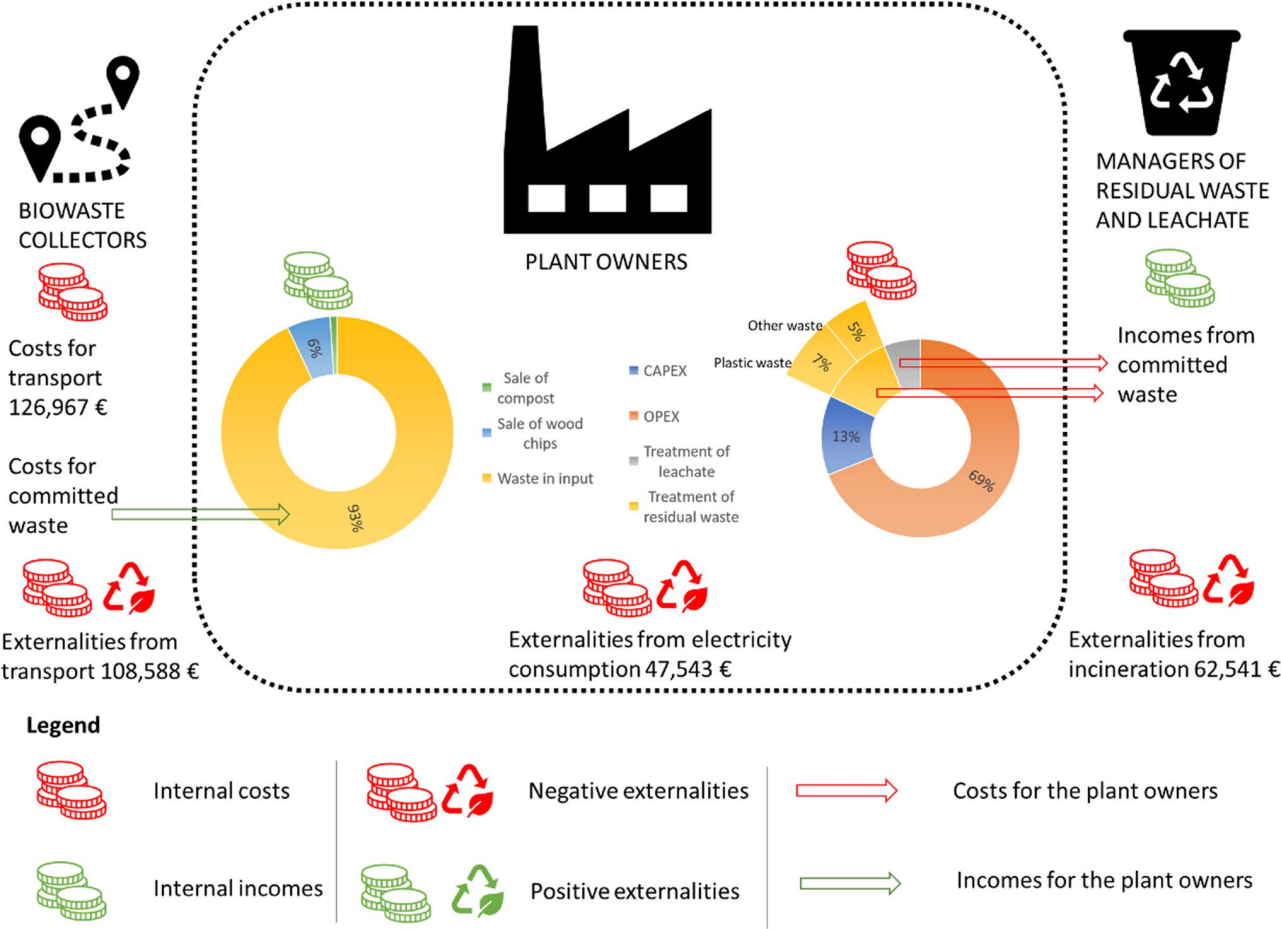
LCC results, including both internal and external costs/incomes, for three actors (biowaste collectors, composting plant owners, managers of residual waste, and leachate). The dotted line encloses the composting plant (own representation)

The results of the LCC analysis are shown in Fig. 5, which displays both internal costs/incomes and positive/negative externalities. For confidentiality reasons, the internal cost and income items of the plant are reported only as a percentage of the total value. As far as internal costs covered by the plant owners are concerned, the operating expenses (OPEX) are the main contributor (69% of total costs), as they include a wide range of expenses, e.g., electricity bills, fuel consumption, and labor costs. It is worth noticing that the machinery in the plant works at an intensive energy rate all over the year. Capital costs (CAPEX) account for 13% of the total costs. The cost for the treatment of residual waste (12% of the total costs) is mostly related to the incineration of plastic waste. The main source of income for the composting plant is the contribution paid by waste collectors for the committed waste (93%), while the sale of compost is not very profitable, as it represents only 1% of the total income. The sale of wood chips accounts for 6% of the total income.

When it comes to externalities, the biggest contribution is given by transport, which reports a negative value of externalities equal to 108,588 €, followed by the negative externalities generated by the electricity consumption of the plant (47,543 €). The positive externalities related to the generation of electricity from the incineration of waste are offset by the negative externalities imputable to the emissions from incineration, meaning that the total externalities from incineration are negative (62,541 €).

**Fig. 6:**
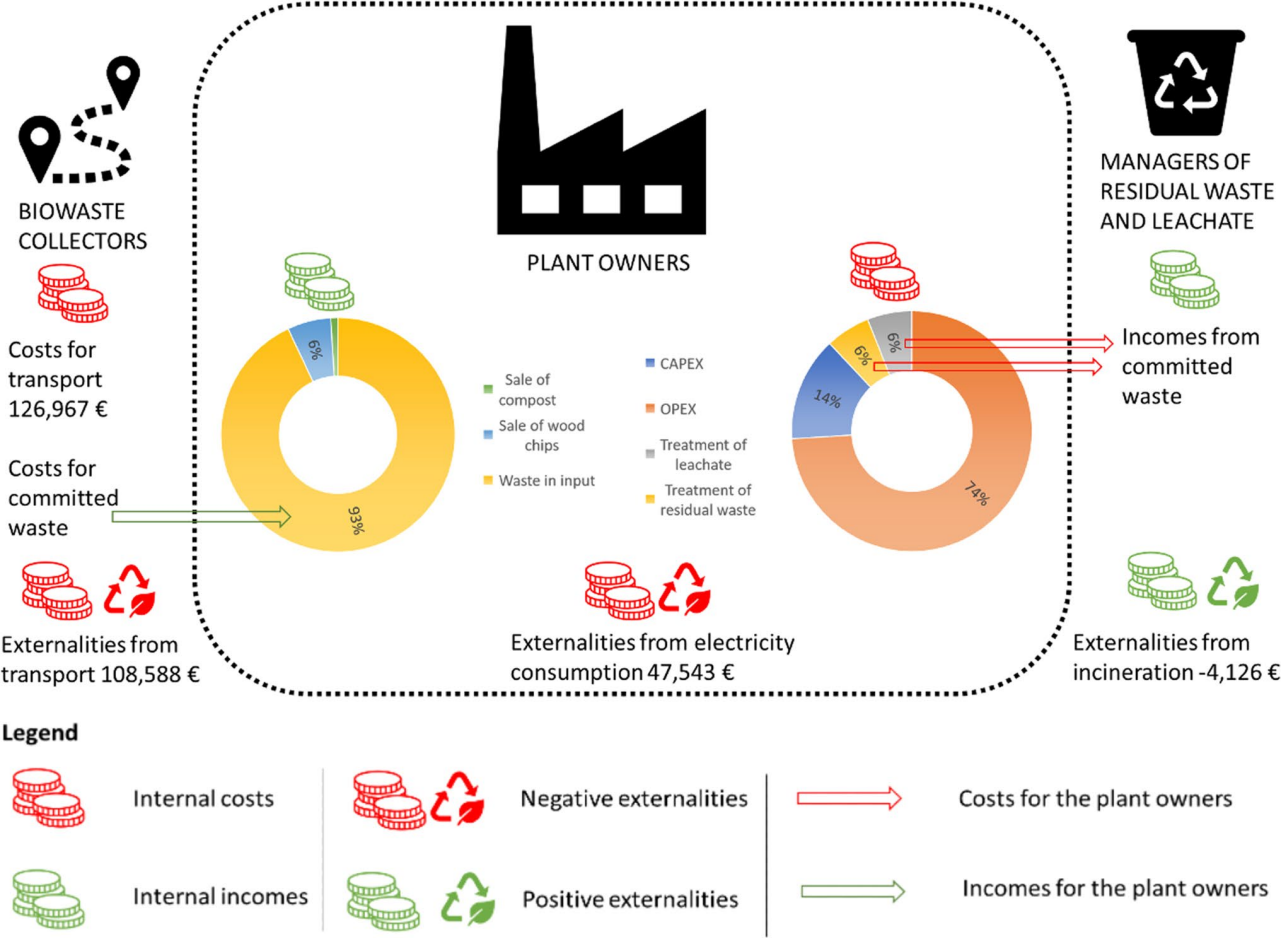
LCC result, scenario analysis, including both internal and external costs/incomes, for three actors (biowaste collectors, composting plant owners, managers of residual waste and leachate). The dotted line encloses the composting plant (own representation)

In the scenario analysis (Fig. 6), the incomes remain unchanged, while the cost structure shows slight variations, due to the fact that a reduction of the costs covered by the plant owner for the treatment of residual waste is expected. All externalities keep the same value as in the baseline scenario, except for externalities from incineration, which become positive (− 4126 €), but still negligible if compared to the negative externalities related to transport and electricity consumption of the plant, which together sum up to 156,131 €. More details are reported in SI.

The results of the LCC are not easy to be compared with other studies in the literature. Indeed, some authors set different system boundaries (e.g., a whole municipal solid waste management system, (Xocaira Paes et al. 2020), or the whole waste management system of a county or region (Martinez-Sanchez et al. 2017; Magrini et al. 2022)), or followed a different methodology to calculate the externalities: for example, Rajabi Hamedani et al. (2020), which assessed an anaerobic digestion power plant, converted the environmental impact (kg CO2 only) to monetary values to internalize the social, ethical, and political cost of this bioenergy system within the economic analysis. On the other hand, Martinez-Sanchez et al. (2017) and Edwards et al. (2018) adopted an emission-based approach and did not highlight the contributions of the various processes (i.e., transportation, electricity consumption, and treatment of residual waste) to the final result. Transport was the main externality also in Magrini et al. (2022), while in that case, incineration of municipal solid waste generated a net external benefit. This difference is due to the waste flows considered: in the case of plastic waste, the avoided emissions are lower than the emissions caused by the incineration process.

## Conclusion

Composting fully embodies sustainability and circular bioeconomy principles by transforming organic waste into nutrient-rich soil amendments that can enhance agricultural productivity, mitigate greenhouse gas emissions, reduce the amount of waste to be landfilled and lower potential fertilizers cost.

Particularly, this research focuses on an Italian composting facility located in the Emilia-Romagna region. The aim was to analyze whether the presence of plastics in biowaste would affect the quality of the final compost and generate negative environmental and economic consequences.

To this end, the study was divided into three sections. A waste flow analysis was first developed by applying the quartering method described in the UNI EN ISO 5667–13 (2000) to quantify and compare the amount of conventional plastic and compostable plastic impurities before and after the composting treatment. The comparison confirmed that conventional plastics and compostable plastic show the opposite behavior during the process. On the one hand, the percentage of conventional plastics remains nearly constant, whereas, on the other hand, the amount of compostable plastic almost disappeared in the composted waste fraction.

Additionally, an environmental life cycle assessment (LCA) and a complementary life cycle costing (LCC) were developed. The two analyses showed consistent outcomes in which the mixing and shredding phases represent the most environmentally demanding stages of the process. In line with this, a similar result was also provided by the economic assessment, in which OPEX is the first contributor to the total annual expenses of the company.

Moreover, a further analysis was developed to investigate better the impacts imputable to the presence of plastic impurities. To this end, a life cycle comparison was conducted between the current situation with an ideal scenario in which no conventional plastic products were present in the collected biowaste, but, instead, plastic impurities consisted solely of compostable plastics. The results show important environmental differences in most of the impact categories analyzed. Conventional plastic impurities mainly influence human, marine, and terrestrial ecotoxicity. These impacts can respectively reduce by 84.5%, 92.6%, and 87.3% if no conventional plastic needs to be treated. It was also proved that plastic impurities are responsible for 7% of the total annual costs covered by the plant owners and around 30% of all the negative externalities. These impacts could be potentially avoided if compostable plastic would entirely replace conventional one.

However, the results partially describe the degree of danger that conventional plastic particles represent when present in the organic fraction. Additional investigations and contributions may be necessary to further extend the analysis outside the boundaries selected, including the use phase of the compost. For instance, Do Carmo Precci Lopes et al. (2019) and De Souza Machado et al. (2017) stated that plastic particles negatively affect the ecosystems and human health when present in the compost and therefore used as fertilizers.

To conclude, this study fits into the current Italian context in which compostable plastic is experiencing significant momentum, representing the primary transitional strategy adopted by the country to reduce plastic pollution (Imbert et al. 2019; Imbert 2017).

On another note, the present results confirm the importance of improving waste separation at the source. The intended achievement can wisely be addressed by raising citizens’ awareness and intensifying communication strategies regarding the consequences and damages of mismanaged waste to the environment and human health.

### Supplementary Information

Below is the link to the electronic supplementary material.

Supplementary file1 (DOCX 57 KB)

## Data Availability

Datasets used and/or analyzed during the current study are available from the corresponding author upon reasonable request.
