# Supplementary material for: Plastic impurities in biowaste treatment: environmental and economic life cycle assessment of a composting plant
Source: Environ Sci Pollut Res Int. 2023 Jul 5;31(7):9964–80. doi: 10.1007/s11356-023-28353-8 (PMC10850183; doi:10.1007/s11356-023-28353-8)
Supplement: Supplementary file 1 — Supplementary file1 (DOCX 57 KB) [file 11356_2023_28353_MOESM1_ESM.docx]

# Supplementary information

**Plastic impurities in biowaste treatment: environmental and economic life cycle assessment of a composting plant**

Sara Bottausci^1^, Chiara Magrini^1^, Giulia Adele Tuci^2^, Alessandra Bonoli^1^

^1^Department of Civil, Chemical, Environmental and Materials Engineering, University of Bologna, Bologna, 40131, Italy

^2^Department of Environmental Science, Informatics and Statistics, University of Venice Ca’ Foscari, Venice Mestre, 30172, Italy

Keywords: compostable plastic, waste management, life cycle costing, material flow analysis, food waste composting plant Corresponding author email: [sara.bottausci2@unibo.it](mailto:sara.bottausci2@unibo.it)

The research paper analyses the plant of the Cooperative “La Città Verde”, which aims to provide quality services and fair products in different fields: landscaping, waste management and recovery, installation and maintenance of urban furniture and social agriculture. Complementarily, the cooperative seeks to create also a sustainable social space by generating work opportunities and integrating people in need.

# **“La Città Verde” composting plant**

The municipalities serviced by La Città Verde composting plant are grouped in Table S1.

**Table S1** List of municipalities and relative distanced from La Città Verde

| **Location** | **Distance to the plant** |
| --- | --- |
| CENTO (FE) | 7,3 km |
| CREVALCORE (BO) | 11 km |
| TERRE DEL RENO (FE) | 16,2 km |
| APRILIA (LT) | 462 km |
| GAGGIO MONTANO (BO) | 92,1 km |
| FANO (PU) | 205 km |
| SANT'AGATA BOLOGNESE (BO) | 19,7 km |
| POGGIO RENATICO (FE) | 26,5 km |
| VIGARANO MAINARDA (FE) | 26,7 km |
| PONTEDERA (PI) | 177 km |
| RAVARINO (MO) | 15,5 km |
| BOLOGNA | 42,8 km |
| SAN PIETRO IN CASALE (BO) | 19,3 km |
| SASSO MARCONI (BO) | 48,5 km |
| BENTIVOGLIO (BO) | 26,5 km |
| NONANTOLA (MO) | 24,3 km |
| SALA BOLOGNESE (BO) | 21,3 km |
| MODENA | 36,8 km |
| SESTO DI RASTIGNANO (BO) | 55 km |
| PIEVE DI CENTO (BO) | 10,5 km |
| IMOLA (BO) | 81,7 km |
| SANT'AGOSTINO (FE) | 15,6 km |
| CARPI (MO) | 38,1 km |
| SAN BENEDETTO DEL TRONTO (AP) | 333 km |
| ANZOLA DELL'EMILIA (BO) | 28,3 km |
| CASTELLO D'ARGILE (BO) | 14,3 km |
| BONDENO (FE) | 25 km |
| CASALFIUMANESE (BO) | 95 km |
| BOMPORTO (MO) | 20,8 km |
| CASTEL MAGGIORE (BO) | 33,3 km |
| SAN GIOVANNI IN PERSICETO (BO) | 15,1 km |
| VILLANOVA DI CASTENASO (BO) | 47 km |
| SAN LAZZARO DI SAVENA (BO) | 48,9 km |
| FERRARA | 41,9 km |
| MONTESE (MO) | 81 km |
| FINALE EMILIA (MO) | 11,8 km |
| CASTELFRANCO EMILIA (MO) | 30,4 km |
| MONTE SAN PIETRO (BO) | 48,9 km |
| SAN GIORGIO DI PIANO (BO) | 23,6 km |
| VALSAMOGGIA (BO) | 40,2km |
| CASTENASO (BO) | 47,1 km |
| ARGELATO (BO) | 20 km |
| ZOLA PREDOSA (BO) | 40,5 km |
| SAN PROSPERO (MO) | 25 km |
| MEDOLLA (MO) | 22,5 km |
| SAN MATTEO DELLA DECIMA (BO) | 6,6 km |
| CALDERARA DI RENO (BO) | 28,5 km |
| CASALECCHIO DI RENO (BO) | 37,6 km |
| CASTEL SAN PIETRO TERME (BO) | 69,6 km |

**Table S2** List of municipalities served by La Città Verde

| **Location municipalities** | | |
| --- | --- | --- |
| Ancarano (TE) | Bondeno (FE) | Castelfranco Emilia (MO) |
| Anzola Emilia (BO) | Budrio (BO) | Castello d’argile (BO) |
| Argelato (BO) | Cadriano (BO) | Cento FE |
| Argenta (FE) | Calderara di reno (BO) | Crevalcore (BO) |
| Battipaglia (SA) | Carpi (MO) | Ferrara fe - finale Emilia (MO) |
| Bentivoglio (BO) | Casalecchio di reno (BO) | Galliera (BO) |
| Bologna (BO) | castel maggiore (BO) | Genzano di Lucania (PZ) |
| Granarolo dell’Emilia (BO) | Imola (BO) | Malalbergo (BO) |
| Marzabotto (BO) | Minerbio (BO) | mirandola (MO) |
| Modena (MO) | Molinella (BO) | Nonantola (MO) |
| Osimo (AN) | Palata Pepoli (BO) | Pietrasanta LU |
| pieve di cento (BO) | Poggio Renatico (FE) | Portomaggiore (FE) |
| Ravarino (MO) | Ravenna (RA) | Rolo (RE) |
| San Benedetto del Tronto (AP) | San Lazzaro di Savena (BO) | San Pietro in Casale (BO) |
| Sant’Agata Bolognese (BO) | S. Maria Codifiume (FE) | Sala bolognese (BO) |
| San Giovanni in Persiceto (BO) | Sasso Marconi (BO) | sassuolo (MO) |
| Sesto di Rastignano (BO) | Tarquinia (VT) | Terre del reno (FE) |
| Valsa moggia (BO) | Vescovana (PD) | Vigarano Mainarda (FE) |
| Villanova di Castenaso (BO) | Zola Predosa (BO) |  |

# **Life cycle assessment (LCA)**

The CML-IA baseline was the methodology used for the assessment and the impact categories included in the calculation are shown in Table S3.

**Table S3** Impact categories of CML-IA methodology

| Abiotic depletion | kg Sb eq |
| --- | --- |
| Abiotic depletion (fossil fuels) | MJ |
| Global warming (GWP100a) | kg CO2 eq |
| Ozone layer depletion (ODP) | kg CFC - 11 eq |
| Human toxicity | kg 1,4 - DB eq |
| Fresh water aquatic ecotox. | kg 1,4 - DB eq |
| Marine aquatic ecotoxicity | kg 1,4 - DB eq |
| Terrestrial ecotoxicity | kg 1,4 - DB eq |
| Photochemical oxidation | kg C_2_H_4_ eq |
| Acidification | kg SO_2_ eq |
| Eutrophication | kg PO_4_ eq |

## **Life Cycle Inventory (LCI)**

As far as the modelling of transport is concerned, an Iveco Stralis 2012 of 12,5 tons of maximum capacity is used as a transportation means for collecting the waste. The transport calculation was performed considering the closest lorry available in the EcoInvent database to the real case, taking also into consideration the weight of the vehicle. The maximum capacity was assumed for every travel; the distances imputable to the collection of organic waste and wood waste were summed together to calculate the total amount of km. The inventory was calculated for one year according to the FU.

**Table S4** Detailed processes for LCI – secondary data sources (Corresponding to Table 1 and Table 2 of the manuscript)

| Element | Data source – Ecoinvent process |
| --- | --- |
| Transportation | |
| **Input** |  |
| Lorry 16 – 32 t | Transport, freight, lorry 16-32 metric ton, EURO4 {RER}\| transport, freight, lorry 16-32 metric ton, EURO4 \| Cut-off, S |
| Shredding mixing and maturation | |
| **Input** |  |
| Mechanical shovels | Diesel {Europe without Switzerland}\| market for \| Cut-off, S |
| Mixer | Electricity, medium voltage {IT}\| market for \| Cut-off, S |
| Aspirator | Electricity, medium voltage {IT}\| market for \| Cut-off, S |
| **Output** |  |
| Leachate to wastewater treatment | Wastewater to treatment –  Details of the Ecoinvent process: Wastewater purified in a smaller municipal wastewater treatment plant (capacity class 4), with an average capacity size of 5320 per-capita-equivalents PCE.  Wastewater contains (in kg/m3): COD^^[[1]](#footnote-1)^^: 11.69 (GSD^^[[2]](#footnote-2)^^=122.5%) |
| Screening & waste management | |
| **Input** |  |
| Screener | Electricity, medium voltage {IT}\| market for \| Cut-off, S |
| **Output** |  |
| Metal waste treatment | Scrap aluminium {RoW}\| treatment of, municipal incineration \| Cut-off, S |
| Glass waste treatment | Waste glass {RoW}\| treatment of waste glass, municipal incineration \| Cut-off, S |
| Textile waste treatment | Waste textile, soiled {RoW}\| treatment of, municipal incineration \| Cut-off, S |
| Plastic mixture waste treatment | Waste plastic, mixture {RoW}\| treatment of waste plastic, mixture, municipal incineration \| Cut-off, S |

## **Scenario Analysis. Waste treatment comparison**

A comparison analysis was developed between the current scenario - *as-is -* in which plastic products are present in biowaste and consequently they undertake a treatment after the composting process, with an ideal scenario - *to-be -* in which only biodegradable plastic was assumed present in the collected biowaste and, therefore, able to degrade and to turn into compost at the end of the treatment process. Table S5 shows the impacts imputable to the waste treatment in the two scenarios, as well as the percentage of reduction, for each impact category.

**Table S5** Variation of the impact of waste treatment in the two scenarios analysed

| **Comparison** | | | |
| --- | --- | --- | --- |
| **Label** | **Waste treatment – As is** | **Waste treatment – To be** | **% Reduction** |
| Abiotic depletion | 24.72 | 15.49 | 37.32 |
| Abiotic depletion (fossil fuels) | 10.15 | 6.69 | 34.03 |
| Global warming (GWP100a) | 80.39 | 31.89 | 60.33 |
| Ozone layer depletion (ODP) | 9.60 | 5.92 | 38.34 |
| Human toxicity | 86.25 | 13.36 | 84.51 |
| Fresh water aquatic ecotox. | 92.57 | 22.46 | 75.73 |
| Marine aquatic ecotoxicity | 77.77 | 5.79 | 92.56 |
| Terrestrial ecotoxicity | 37.02 | 4.69 | 87.33 |
| Photochemical oxidation | 13.90 | 9.41 | 32.31 |
| Acidification | 22.98 | 16.87 | 26.59 |
| Eutrophication | 26.27 | 18.40 | 29.96 |

# **Life cycle cost (LCC)**

## **Costs of transport**

The total transportation cost was calculated referring to 2020, considering the average annual fuel cost, the fuel consumption cost of the considered Iveco Stralis 2012, the highway tolls cost and the cost of personnel. Potential fines or rental costs were not considered in the calculation.

Similar to the LCA calculation set up, the transportation cost was also conducted considering different travels back and forwards from the plant facility to the collection sites according to the distance and to the total amount of waste to be collected. Organic waste and wood waste are not collected in the same sites and, this time, two different transport means having both 12,5 tons of maximum load capacity were assumed in the calculation. Collection locations accounting for less than 12,5 tons were added together to maximize the transportation loads. This approximation was reasonably appropriate for the purpose of the calculation, as they do not particularly influence the result contributing only for 1,15 % of the total amount of the waste collected.

## **Externalities from transport**

The main reference for the calculation of externalities from transport was the “Handbook on the external costs of transport” authored by CE Delft (2019). Table S6 shows the values referred to a vehicle within the class EURO 3, diesel, 7,5-16 t (fuel inefficient HGVs: 716 g/km).

Table S6 Marginal cost [€-cent/tkm] for the externalities from transport

|  | €-cent/tkm | | |
| --- | --- | --- | --- |
|  | Marginal cost for dense metropolitan traffic during the day | Marginal cost for dense traffic on rural motorways during the day | Average |
| Accident | 0.1 | 0.07 | 0.085 |
| Air pollution | 1.61 | 0.5 | 1.055 |
| Climate change | 0.83 | 0.58 | 0.705 |
| Noise | 0.7 | 0.01 | 0.355 |
| Congestion | 9.7 | 4.3 | 7 |
| Waste to tank^^[[3]](#footnote-3)^^ | 0.19 | 0.14 | 0.165 |
| **Total** |  |  | **9.365** |

## **Externalities from electricity consumption**

Energy consumption (and the related emissions) was considered as the main source of externality for the plant. The plant uses 728,640 KWh every year. It is assumed that the energy consumption is the same in both the scenarios. As far as the external cost of pollutants is concerned, following Magrini et al. (2021), the valuations proposed by CE Delft (2018) were adopted, complemented with a “proxy” value for HCl and HF obtained by rescaling that of SO2 according to their respective recommended emission limits in the recent European Best Available Technology reference document on waste incineration (Neuwahl et al., 2019). The unit costs are shown in Table S7.

Table S7- External cost of pollutants

| **Reference** | **External cost of pollutant [€/kg]** | | | | | | | | |
| --- | --- | --- | --- | --- | --- | --- | --- | --- | --- |
|  | **PM** | **Nox** | **SOx** | **HCl** | **HF** | **CO** | **PCDD** | **Hg** | **CO_2_** |
| CE Delft (2018) | 38.7 | 14.8 | 11.5 |  |  | 0.05 | 1810 | 34500 | 0.057 |
| Magrini et al., 2021 |  |  |  | 57.5 | 575.5 |  |  |  |  |

The emission factor of the Italian grid mix was considered, as shown in Table S8.

Table S8- Emission factors for electricity generation, Italian grid mix

| **Pollutant** | **Emission factors (kg/kWh)** |
| --- | --- |
|  | Electricity generation, Italian energy mix (EMEP, 2017) |
| CO_2_ | 1.110 |
| SO*_2_* | 2.99E-05 |
| NO_2_ | 1.03E-04 |
| PM | 1.25E-06 |
| CO | 6.93E-05 |
| Hg | 1.69E-09 |
| PCDD | 8.57E-15 |

## **Externalities from incinerators**

The analysis was based on the study performed by Magrini et al. (2021), including the external costs from macropollutants and external benefits related to the avoided electricity production, considering again the Italian energy mix. The average value of electricity generation (Table S9) was calculated, starting from the data of the plants located in the Region.

Table S9- Incinerators in the Emilia-Romagna region: electricity generated per ton of treated waste

| Incineration plants in Emilia-Romagna Region | Electricity generated [MWh/t] |
| --- | --- |
| FE-INC (HERAmbiente Ferrara) | 0.395134018 |
| FC-INC (HERAmbiente Forlì) | 0.512613589 |
| RN-INC (HERAmbiente Rimini) | 0.467967763 |
| BO-INC (FEA Bologna) | 0.530997541 |
| MO-INC (HERAmbiente Modena) | 0.588344625 |
| WTE IREN Parma | 0.52074744 |
| PC-INC (Tecnoborgo Piacenza) | 0.640456848 |
| wte Heramb (HERAmbiente Ravenna) | 0.752064761 |
| **Regional average** | 0.551040823 |

The electricity generated from the plastic waste and from the other waste in output to the plant are shown in Table S10, for both the scenarios.

Table S10- Electricity generated from waste treatment

|  | Electricity [Kwh], baseline | Electricity [Kwh], scenario |
| --- | --- | --- |
| Electricity from plastic waste | 330,624 | - |
| Electricity from other waste | 394,270 | 394,270 |
| Total value of electricity from incineration of waste | 724,894 | 394,270 |

In particular, the values of the emissions of macropollutants available in SimaPro were gathered (Table S11).

All the main macropollutants generated by waste incineration were included in the analysis: acid gases (SO2, HCl and HF), CO, NOx, and particulate matter. In addition, the analysis included the two micropollutants for which monitoring of the annual mass flow is mandatory for WtE plants in the Region: Hg and the class of polychlorinated dibenzo-p-dioxins and dibenzofurans (PCDD/Fs).

Table S11 - Emissions from incinerators

|  | PM | NOx | HCl | HF | SO2 | CO | PCDD | Hg | Fossil  CO2 |
| --- | --- | --- | --- | --- | --- | --- | --- | --- | --- |
| Amount generated [Kg] | 6.30E+01 | 1.15E+03 | 8.19E+00 | 2.88E-01 | 1.29E+02 | 1.77E+02 | 6.87E-09 | 1.52E-02 | 1.53E+06 |
| Amount generated [Kg], scenario | 3.71E+01 | 791 | 1.22E+00 | 0.117 | 85.2 | 95.6 | 6.57E-03 | 8.99E-04 | 115000 |

# **References**

CE Delft, 2018. Environmental Prices Handbook, EU28 version.

CE Delft, 2019. Handbook on estimation of external costs in the transport sector. Luxembourg, January 2019

Di Maria, F., Micale, C., 2015. Life cycle analysis of management options for organic waste collected in an urban area. Environ. Sci. Pollut. Res. 22, 248–263. https://doi. org/10.1007/s11356-014-3330-9.

Di Maria, F., Micale, C., Contini, S., Morettini, E., 2016. Impact of biological treatments of bio-waste for nutrients, energy and bio-methane recovery in a life cycle perspective. Waste Manag. 52, 86–95. https://doi.org/10.1016/j. wasman.2016.04.009.

EMEP, 2017. The Emissions Database. European Monitoring and Evaluation Programme. Available at: <https://www.ceip.at/webdab-emission-database>

Lombardi, L., Carnevale, E.A., Corti, A., 2015. Comparison of different biological treatment scenarios for the organic fraction of municipal solid waste. Int. J. Environ. Sci. Technol. 12, 1–14. https://doi.org/10.1007/s13762-013-0421-y.

Magrini, C., Dal Pozzo, A., Bonoli, A., Assessing the externalities of a waste management system via life cycle costing: the case study of the Emilia-Romagna Region (Italy), «Waste management», 2021, under review

Mancini, E., Arzoumanidis, I., Raggi, A., 2019. Evaluation of potential environmental impacts related to two organic waste treatment options in Italy. J. Clean. Prod. 214, 927–938. https://doi.org/10.1016/j.jclepro.2018.12.321.

Neuwahl, F., Cusano, G., Benavides, J.G., Holbrook, S., Roudier; S., Best Available Techniques (BAT) Reference Document for Waste Incineration; EUR 29971 EN;doi:10.2760/761437"

Sailer G, Eichermüller J, Poetsch J, Paczkowski S, Pelz S, Oechsner H, Müller J, Characterization of the separately collected organic fraction of municipal solid waste (OFMSW) from rural and urban districts for a one-year period in Germany (2021) Waste Management <https://doi.org/10.1016/j.wasman.2021.07.004> - p. 471–482

Saer, A., Lansing, S., Davitt, N.H., Graves, R.E., 2013. Life cycle assessment of a food waste composting system: environmental impact hotspots. J. Clean. Prod. 52, 234–244. https://doi.org/10.1016/j.jclepro.2013.03.022.

Salemdeeb, R., Daina, M.B., Reynolds, C., Al-Tabbaa, A., 2018. An environmental evaluation of food waste downstream management options: a hybrid LCA approach. Int. J. Recycl. Org. Waste Agric. 7, 217–229. https://doi.org/10.1007/s40093-018- 0208-8.

Slorach, P.C., Jeswani, H.K., Cu ́ellar-Franca, R., Azapagic, A., 2019. Environmental and economic implications of recovering resources from food waste in a circular economy. Sci. Total Environ. 693, 133516. https://doi.org/10.1016/j. scitotenv.2019.07.322.

Thyberg, K.L., Tonjes, D.J., 2017. The environmental impacts of alternative food waste treatment technologies in the US. J. Clean. Prod. 158, 101–108. https://doi.org/ 10.1016/j.jclepro.2017.04.169.

Tonini, D., Wandl, A., Meister, K., Unceta, P.M., Taelman, S.E., Sanjuan-Delm ́as, D., Dewulf, J., Huygens, D., 2020. Quantitative sustainability assessment of household food waste management in the Amsterdam Metropolitan Area. Resour. Conserv. Recycl. 160, 104854. https://doi.org/10.1016/j.resconrec.2020.104854.

1. COD = Chemical oxygen demand [↑](#footnote-ref-1)
2. GSD = Reverse Flow Disintegration [↑](#footnote-ref-2)
3. According to CE DELFT (2019), the cost of well-to-tank emissions (= costs of energy production) includes the production of all different type of energy sources which leads to emissions and other externalities (i.e., the extraction of energy sources, the processing, the transport and transmission, the building of energy plants and other infrastructures). [↑](#footnote-ref-3)
